# Early and mid-term results with the ATTUNE total knee replacement system compared to PFC Sigma: a prospective comparative study

**DOI:** 10.1186/s13018-022-03397-7

**Published:** 2022-11-24

**Authors:** Roland E. Willburger, Stella Oberberg

**Affiliations:** Department of Orthopaedic Surgery, Ruhr University Bochum, Katholisches Klinikum Bochum, Bochum, Germany

**Keywords:** Total knee arthroplasty, ATTUNE, PFC Sigma, PROMs, Radiolucent lines, Outcome

## Abstract

**Purpose:**

Up to 20% of all patients are not satisfied with the result after total knee arthroplasty (TKA). To improve patient satisfaction manufacturers have modified prosthesis design. The ATTUNE prosthesis is a modified version of the PFC Sigma. Aim of this study was to evaluate the outcome at 6 months and 5 years after TKA with ATTUNE compared to PFC Sigma.

**Methods:**

Sixty patients were included prospectively (30 ATTUNE vs. 30 PFC Sigma). Knee Society Score and Hospital for Special Surgery Score were recorded preoperatively, at 6 months and at least 5 years postoperatively. At 5-years follow-up X-rays in two planes were evaluated, radiolucent lines were documented.

**Results:**

Patient characteristics were similar in both groups. Both ATTUNE and PFC Sigma provided good to excellent clinical results. There were no statistically significant differences based on the overall scores and patient rated outcome measures. Nevertheless, patients in the ATTUNE group tended to be symptom-free earlier and to achieve better clinical results after 5 years.

**Conclusion:**

Even with the scores not being significantly different here, the modified design of ATTUNE could increase long-term satisfaction with the implant and reduce the need for revision surgery. However, long-term results are required to prove this.

## Background

Total knee arthroplasty (TKA) is one of the most common and successful surgeries with more than 95 percent implant survivorship at 10 years [9]. The aim of TKA is to relieve pain and improve joint functionality and patient's quality of life. Despite very good long-term survivorship, there are some patients showing persistent limitations and pain. Up to 20% of patients are unable to achieve the expected functional outcome and are dissatisfied after TKA [2, 3]. To improve patient satisfaction with TKA manufacturers have modified their prosthesis designs. The ATTUNE prosthesis (Depuy Synthes) is a modified version of the PFC Sigma (Depuy Synthes). The theoretical advantages of the ATTUNE prosthesis are increased conformity between the femoral component and tibial polyethylene insert, more prosthesis sizes for individual adjustment, optimization of patellofemoral conformity, and an improved polyethylene insert locking mechanism [4]. These theoretical advantages should improve patient satisfaction and joint stability, reduce persistent pain and therefore optimize outcome of TKA.

Aim of this study was to evaluate the outcome (including PROMs) at 6 months and at least 5 years after TKA with ATTUNE compared to PFC Sigma.

## Methods

### Patients

Sixty patients were included prospectively in the study and alternately assigned to ATTUNE and PFC Sigma over a period of 18 months. Thus 30 cemented, cruciate retaining, fixed bearing, primary ATTUNE TKAs (30 patients) were compared to 30 cemented, cruciate retaining, fixed bearing, primary PFC Sigma TKAs (30 patients).

*Inclusion criteria* Age 70 or younger and no severe health problems—so that long-term follow-up (FU) could be expected. Osteoarthritis with indication for total knee replacement, intact collateral ligaments and posterior cruciate ligament.

*Exclusion criteria* Previous surgery on the joint with the exception of arthroscopy (no cruciate ligament repair), lack of commitment to participate in the FU examinations, mental or linguistic deficits that impair a meaningful survey.

### Surgery

Two experienced surgeons used identical surgical techniques to implant both designs. Both surgeons had many years of experience with the PFC Sigma knee system and were trained to use the ATTUNE knee system. Prior to starting the study, each operated on ten patients using the ATTUNE knee system to obtain experience and confidence with the design and instrumentation. Surgery was performed under general or regional anesthesia. A standard midline skin incision with a medial parapatellar arthrotomy and standard bone referenced measured resection balancing techniques were used. The posterior cruciate ligament was always retained and a tourniquet used. In all patients a patella resection arthroplasty and no patella resurfacing were performed.

### Follow-up treatment

Mobilization with physiotherapy and full weight bearing (using 2 crutches) began the first day after surgery. After discharge from the hospital an outpatient or inpatient rehabilitation was carried out.

### Data collection

The Knee Society Score (KSS) and Hospital for Special Surgery Score (HSS) were recorded preoperatively and during the FU examinations at 6 months and at least 5 years postoperatively [7, 10]. During the 5-year FU current X-ray images of the respective knee joint in two planes were evaluated by two experienced doctors with regard to prosthesis positioning, position changes and visible radiolucent lines (RLL).

### Statistics

All statistical analyses were performed as intention-to-treat with SPSS 23.0 (IBM, Chicago, IL) at a level of significance of 0.05. Metric variables were assessed by Mann–Whitney U test and categorical variables using Fisher exact test. Descriptive statistics are displayed as means with standard deviation or range for continuous variables and frequencies with percentages for categorical variables.

## Results

The 60 patients included had 28 different secondary diagnoses, the most common of which were hypertension followed by diabetes mellitus. These secondary diseases and especially those that could have a possible influence on the ability to walk, such as polyneuropathia or Parkinson's disease, were equally distributed in both groups. The only significant difference between the two groups was the older age in the PFC group. Significantly more women were included; this gender distribution did not change significantly during the observation period (*p* value 0.789). Only one patient could not be re-examined after 6 months but after 5 years.

When the patients were invited by telephone for the five-year FU examination, six patients in the ATTUNE group declined a FU examination. Three of them declined due to serious illnesses (carcinoma, Parkinson's disease, recent heart attack with ongoing rehabilitation). Three patients refused to travel the long distance to our hospital. All of them stated that they were essentially satisfied with the outcome of the TKA and that no reoperation had taken place. Three patients could not be reached either by phone or by mail. One patient had died unrelated to the TKA.

In the PFC group six patients were not reexamined at 5-year FU. One patient was excluded due to reoperation following aseptic loosening of the tibial prosthesis component. One patient had died unrelated to the TKA. Two patients could not be reached by phone or by mail. Two patients were not examined because they had intermittently acquired dementia and Parkinson's disease, respectively. In both cases, however, we were informed that there were no relevant problems concerning the knee prosthesis and that no revision surgery had been performed.

Since the FU rate was approximately the same after 5 years in both groups a comparison was possible.

The frequency of complaints regarding other joints (*p *value 0.057) that are relevant for walking, as well as the leg axes (*p *value 1.0) were not significantly different preoperatively (Tables 1, 2, 3, 4 and 5).

**Table 1:** Clinical data preoperatively and at follow-up (FU)

Clinical date	Preoperatively		6 month FU		5-year FU	
Implant	ATTUNE	PFC	ATTUNE	PFC	ATTUNE	PFC
Implant (*n* =)	30	30	29	30	20	24
Age at surgery (years)	58.6	63.5			64.3	69.6
Females (*n* =)	20 (67%)	18 (60%)	19 (65%)	18 (60%)	14 (70%)	14 (58%)
Affected knee right/left	17/13	20/10	16/13	20/10	12/8	17/7
Complaints contralateral knee	8 (27%)	2 (7%)	7 (24%)	2 (7%)	5 (25%)	7 (29%)
Hip discomfort	1 (3%)	1 (3%)	1 (3%)	1 (3%)	1 (5%)	2 (8%)
No axis deviation (*n* =)	21 (70%)	15 (50%)				
Varus deformity (*n* =)	4 (13%)	9 (30%)				
Valgus deformity (*n* =)	5 (17%)	6 (20%)

**Table 2:** Statistical analysis preoperatively, at 6 month and 5-year follow-up (FU)

Age (years)		*N*	Mean	SD	Median	Min–max	*p* value
Preoperatively	ATTUNE PFC	30 30	58.6 63.5	7.6 3.7	61 65	38–69 55–69	0.004*
5-year FU	ATTUNE PFC	20 24	64.3 69.6	8.8 3.6	66 71	43–76 61–75	0.014*

* means significant difference

**Table 3:** Range of motion preoperatively, at 6 month and 5-year follow-up

ROM (°)		*N*	Mean	SD	Median	Min–max	*p* value
Preoperatively	ATTUNE PFC	30 30	111.2 107.5	14.9 16.3	110 105	70–145 70–135	0.352
6 month FU	ATTUNE PFC	29 30	110.3 110.3	12.6 11.7	110 110	80–130 90–135	0.724
5-year FU	ATTUNE PFC	20 24	117.0 118.3	12.7 10.4	120 118	90–130 100–135	0.952

**Table 4:** KSS Score preoperatively, at 6 month and 5-year follow-up

KSS score (max 200)		*N*	Mean	SD	Median	Min–max	*p* value
Preoperatively	ATTUNE PFC	30 30	105.6 96.5	17.5 26.3	103 100	63–139 44–145	0.249
6 month FU	ATTUNE PFC	29 30	168.9 158.6	22.9 34.0	176 174	104–192 23–192	0.160
5-year FU	ATTUNE PFC	20 24	178.2 170.2	26.6 22.2	189 175	110–200 120–200	0.106

**Table 5:** HSS Score preoperatively, at 6 month and 5-year follow-up

HSS score (max 100)		*N*	Mean	SD	Median	Min–max	*p* value
Preoperatively	ATTUNE PFC	30 30	64.7 63.1	10.4 10.0	65 64	41–82 44–79	0.584
6 month FU	ATTUNE PFC	29 30	85.3 80.7	8.8 13.8	88 86	54–95 28–96	0.194
5-year FU	ATTUNE PFC	20 24	88.9 85.9	10.6 7.9	93 86	65–97 70–98	0.074

Six months after surgery, the following data was collected: numerical pain scales (at rest, at night, when standing, when climbing stairs, when walking), duration of the postoperative use of painkillers, duration of the use of crutches after surgery, maximum possible walking time without a pause, duration of the postoperative knee swelling, ability to take part in sports and the point in time at which this was possible again postoperatively. Here the ATTUNE group showed significantly lower night pain (*p *value 0.041) and also a significantly shorter duration of analgetic medication postoperatively (*p *value 0.049). None of the other data differed significantly.

At the 5-year FU, the results had improved further in all but one patient. To the best of our knowledge, only one of the knee joints (PFC group) had been operated on again due to aseptic loosening of the tibial part of the prosthesis.

The clinical examination at 5-year FU showed no significantly different capsule-ligament loosening in extension (*p *value 0.731), 30° flexion (*p *value 1.0), 60° flexion (*p *value 0.492) and 90° flexion (*p *value 1.0). Anteroposterior stability, manually tested by means of a drawer test, was not significantly different either (*p *value 0.298). Radiolucent lines (RLL) at the implant–cement interface on the tibial side were not significantly different in extent and frequency (*p *value 0.389).

Only one patient in the PFC group showed a femoral RLL of less than 2 mm width in zone 4 according to Ewald [5] (Tables 6 and 7).

**Table 6:** Radiolucent lines (RLL) at the 5-years FU: Tibial component anterior view zones 1–7 according to Ewald [5]

Zone	ATTUNE (*n* = 20)	PFC Sigma (*n* = 24)
1	2 (10%)	5 (21%)
2	6 (30%)	8 (33%)
3	1 (5%)	0 (0%)
4	0	0
5	0	0
6	0	0
7	0	0

**Table 7:** Radiolucent lines (RLL) at the 5 years FU: Tibial component lateral view zones 1–3 according to Ewald [5]

Zone	ATTUNE (*n* = 20)	Pfc Sigma (*n* = 24)
1	2 (10%)	3 (13%)
2	1 (5%)	2 (8%)
3	0	0

## Discussion

Patient reported outcome measures (PROMs) were collected at 6 months and 5 years after surgery using KSS and HSS. Based on these total score results, there was no significant difference between the two groups.

Mid-range instability is attributed to multi-radius femoral components allowing transient ligament slackness during knee flexion [13]. Single radius design has been introduced to avoid this. However, kinematic and stability tests could not demonstrate enhanced mid-range stability of the single-radius TKA over older multi-radius implant [13].

The ATTUNE prosthesis was developed to improve patellofemoral kinematics (the ATTUNE has an anatomic trochlear groove vs. the PFC Sigma with a single radius trochlear groove) as well as mid flexion stability of the knee with an increased congruence between femoral and tibial articulations. This greater congruence might affect the forces on the tibial implant-bone interface [1]. RLL of 1–2 mm between cement and bone may be due to cement contraction or improper cementation and are not considered to pose problems [6]. However, progressive widening of RLLs points to a possible implant loosening. A change in prosthesis position is a sure sign of loosening. The x-ray examination in 2 planes showed no changes in the position of the implants in our both groups. According to the publication of Smith et al. [12] in 195 TKAs with well-fixed femoral components and presumably low wear, 15% of tibiae developed early-onset, non-progressive partial RLLs. Immediate postoperative RLLs can be secondary to surgical technique with either inadequate cement penetration in sclerotic bone or malpositioning of the component relative to the bone cuts. RLLs without clinical symptoms should be analyzed according to their potential cause of development and followed up closely. A two year follow-up by Kaptein et al. [8] showed that the ATTUNE tibial component was not inferior to the PFC Sigma with respect to overall migration, although it showed more RLLs at the implant–cement interface than PFC Sigma. The version of the ATTUNE tibial component examined in the aforementioned study and our study has subsequently undergone modification by the manufacturer. In our investigation, there was no evidence of increased RLLs or loosening in the ATTUNE group. However, long-term results should be awaited in this regard.

Residual pain contributes to the high patient dissatisfaction rate after TKA. Anterior knee pain (AKP) is an important cause of dissatisfaction after TKA. It has been suggested that modifications to the patellofemoral joint may reduce residual AKP. As the ATTUNE was designed to address this issue Ranawat et al. [11] compared the ATTUNE to the PFC Sigma (all implants were cemented, posterior stabilized, and the patella resurfaced). Based on the KSS (Knee Society Score) clinical rating system, excellent clinical results had been achieved in 89.4% and 90.7% of ATTUNE and PFC Sigma TKAs, respectively. The ATTUNE was associated with a reduced incidence of mild-to-moderate AKP compared to PFC Sigma. There were no significant differences in the KSS pain or function scores [11]. In line with this, our study showed a tendency towards less pain postoperatively and at 5-year FU without significant differences in the overall results of the HSS and KSS scores.

Limitations of our study are the lack of blinding and randomization. Therefore, bias cannot be excluded as a confounding variable. However, our study is the first one (to our knowledge) at 5-year FU comparing ATTUNE to PFC Sigma with detailed evaluation of PROMs and radiographs.

In conclusion, both the ATTUNE and PFC Sigma provided good to excellent clinical results at 6 months and 5-year FU. There were no statistically significant differences with regard to the HSS and KSS. Nevertheless, modified designs in TKA have the potential to improve patient satisfaction especially in the long term, where FU examinations are required to assess this. Besides improvement of the prosthesis design, an exact surgical technique (with preservation of the joint line, balanced flexion and extension gap, optimal patellar tracking, best possible ligament balancing) and realistic patient expectation remain a very important key to success in TKA.

## Data Availability

All authors declare that all data and materials support the published claims and comply with field standards.

## Abbreviations

FU: Follow-up
HSS: Hospital for Special Surgery Score
KSS: Knee Society Score
PROM: Patient rated outcome measure
RLL: Radiolucent lines
ROM: Range of motion
TKA: Total knee arthroplasty
